# ColMA‐based bioprinted 3D scaffold allowed to study tenogenic events in human tendon stem cells

**DOI:** 10.1002/btm2.10723

**Published:** 2024-10-30

**Authors:** Giacomo Cortella, Erwin Pavel Lamparelli, Maria Camilla Ciardulli, Joseph Lovecchio, Emanuele Giordano, Nicola Maffulli, Giovanna Della Porta

**Affiliations:** ^1^ Translational NanoMedicine Laboratory, Department of Medicine, Surgery and Dentistry University of Salerno Baronissi SA Italy; ^2^ School of Science and Engineering Reykjavík University Reykjavík Iceland; ^3^ Institute of Biomedical and Neural Engineering Reykjavik University Reykjavík Iceland; ^4^ Laboratory of Cellular and Molecular Engineering “Silvio Cavalcanti”, Department of Electrical, Electronic and Information Engineering “Guglielmo Marconi” (DEI) University of Bologna Cesena FC Italy; ^5^ Advanced Research Center on Electronic Systems (ARCES) University of Bologna Bologna BO Italy; ^6^ School of Pharmacy and Bioengineering Keele University Stoke‐on‐Trent Staffordshire UK; ^7^ Department of Trauma and Orthopaedics, Faculty of Medicine and Psychology Sant'Andrea Hospital, “La Sapienza” University Rome Italy; ^8^ Research Centre for Biomaterials BIONAM Università di Salerno Fisciano SA Italy

**Keywords:** 3D collagen scaffold, extrusion‐based 3D bioprinting, GDF‐5, human tendon stem progenitor cells, perfusion bioreactor system

## Abstract

The advent of bioprinting has enabled the creation of precise three‐dimensional (3D) cell cultures suitable for biomimetic in vitro models. In this study, we developed a novel protocol for 3D printing methacrylated collagen (ColMa, or PhotoCol®) combined with tendon stem/progenitor cells (hTSPCs) derived from human tendon explants. Although pure ColMa has not previously been proposed as a printable hydrogel, this paper outlines a robust and highly reproducible pipeline for bioprinting this material. Indeed, we successfully fabricated a 3D bioengineered scaffold and cultured it for 21 days under perfusion conditions with medium supplemented with growth/differentiation factor‐5 (GDF‐5). This bioprinting pipeline and the culture conditions created an exceptionally favorable 3D environment, enabling the cells to proliferate, exhibit tenogenic behaviors, and produce a new collagen type I matrix, thereby remodeling the surrounding environment. Indeed, over the 21‐day culture period under perfusion condition, tenomodulin expression showed a significant upregulation on day 7, with a 2.3‐fold increase, compared to days 14 and 21. Collagen type I gene expression was upregulated nearly 10‐fold by day 14. This trend was further confirmed by western blot analysis, which revealed a statistically significant difference in tenomodulin expression between day 21 and both day 7 and day 14. For type I collagen, significant differences were observed between day 0 and day 21, as well as between day 0 and day 14, with a *p*‐value of 0.01. These results indicate a progressive over‐expression of type I collagen, reflecting cell differentiation towards a proper tenogenic phenotype. Cytokines, such as IL‐8 and IL‐6, levels peaked at 8566 and 7636 pg/mL, respectively, on day 7, before decreasing to 54 and 46 pg/mL by day 21. Overall, the data suggest that the novel ColMa bioprinting protocol effectively provided a conducive environment for the growth and proper differentiation of hTSPCs, showcasing its potential for studying cell behavior and tenogenic differentiation.

**Translational Impact Statements**

Our research pioneers the use of methacrylated collagen—without the addition of co‐polymers—in 3D bioprinting for tissue engineering applications. By optimizing printing conditions and incorporating human tendon stem/progenitor cells (hTSPCs), we have developed a new 3D bioplotting pipeline and created a biomimetic environment for studying tenogenic processes. The integration of growth differentiation factor‐5 and a perfusion bioreactor further advances this in vitro model. Future directions include bioplotting primary cells from pathological explants to explore impaired events in pathological conditions.

## 1 INTRODUCTION

In the dynamic and evolving landscape of regenerative medicine and tissue engineering, bioprinting has emerged as a promising technology that offers unprecedented precision and control in fabricating three‐dimensional scaffolds enriched with cells and nanocarriers.^1^, ^2^, ^3^ The combination of bio‐inks, bioprinters, and bioreactors represented a multidisciplinary approach in tissue engineering that offered a way to fabricate synthetic extracellular matrices useful as 3D in vitro models mimicking physiological and/or pathological environments.^4^, ^5^, ^6^ Among bioinks, several methacrylated hydrogels are available to assure a fast crosslinking, with high biocompatibility and bioactivity.^7^, ^8^, ^9^ Among these, methacrylated collagen (ColMa) emerges as a promising and versatile candidate, offering distinct advantages that elevate its potential for use in bioprinting applications, especially to resemble musculoskeletal extracellular matrices (ECM).^10^, ^11^, ^12^, ^13^ Methacrylation modification of collagen chains enables rapid photo‐crosslinking and improves collagen's printability, promoting a precise scaffold architecture control during the bioprinting process.^14^, ^15^ It has also reported that ColMa promotes cellular adhesion, proliferation, and differentiation, fostering a microenvironment mimicking in vivo conditions crucial for musculoskeletal tissue regeneration.^14^ Despite its intriguing properties, a scaffold exclusively made from ColMA has not yet been reported. Previous studies have explored the use of ColMA with others co‐polymers in various tissue engineering applications, but a solely ColMA‐based printed scaffold remains unexplored. Our work is unique in its focus on optimizing bioplotting conditions for ColMA scaffolds combined with human tendon stem/progenitor cells (hTSPCs). This research provides a novel platform for studying tenogenic processes under dynamic culture conditions. By optimizing the ColMA bioplotting process, we aim to enhance its potential for bioprinting and expand its applications significantly. To establish ColMA as a suitable bioink and successfully fabricate a 3D bioengineered in vitro model, it is crucial to ensure that cells are effectively dispersed within the matrix.^5^ Seeding primary or stem cells in 3D in vitro models significantly enhances their relevance and ability to mimic physiological conditions. These cells interact with their microenvironment in a way that closely resembles the architecture of native tissue. This setup facilitates complex interactions between cells and their surrounding matrix, including specific cell–matrix interactions. Such interactions can affect various aspects of tissue development, including cell proliferation, differentiation, and ECM remodeling. Ultimately, these dynamics shape the functional properties of the engineered tissue constructs.^15^, ^16^

A distinct cell population identified in human tendons, commonly known as tendon stem/progenitor cells (TSPCs), has recently been described and characterized.^17^, ^18^ Initially recognized for their mesenchymal stem cell (MSC)‐like attributes, such as typical surface antigen expression, self‐renewal capabilities, clonogenicity, and the potential for multidifferentiation, human TSPCs are pivotal players in tendon development, homeostasis, and healing processes. In vitro, hTSPCs express markers associated with tendons and demonstrate the capacity to generate tendon‐like and enthesis‐like tissues.^18^ Given that existing treatment approaches for tendon disorders often fall short of delivering optimal outcomes,^19^, ^20^, ^21^ the exploration and characterization of hTSPCs present an intriguing avenue for unveiling the distinctive attributes of tendon tissues. To address the differentiation of hTSPCs the use of a growth factor (GF) was described because they can play crucial roles in tissue repair and tenogenic events commitment.^22^ Among them, growth/differentiation factor‐5 (GDF‐5) stands out for its ability to induce the expression of genes associated with the neo‐tendon phenotype.^23^, ^24^ It has been shown by Ciardulli et al.^23^ that GDF‐5 was able not only to promote the expression of tenogenic markers in human bone marrow‐mesenchymal stem cells (hBM‐MSCs) but also in those derived by Wharton's Jelly (hWJ‐MSCs). Specifically, the concentration of 100 ng/mL resulted in the greatest tendon markers expression.^24^, ^25^, ^26^, ^27^, ^28^

Tridimensional culture also requires dynamic environment assured by specific bioreactors to promote active mass transport within the 3D environment.^29^ Perfusion bioreactors can provide constant medium flow^30^, ^31^ through the culture^29^ and successfully assure adequate gas and metabolite exchanges.^32^

Given that the 3D bioprinting of pure ColMa scaffolds is not widely documented, the primary goal of this study was to develop an effective, robust, and reproducible workflow for producing a homogeneous, printable, and cell‐compatible master mix. PhotoCol® is not typically listed as a bioprintable solution and is usually intended for manual extrusion; this work aimed to overcome this limitation and expand its potential applications. Additionally, the ultimate objective was to fabricate bioengineered collagen 3D scaffolds to facilitate the study of tenogenic events in hTSPCs. To achieve this, we employed a perfusion bioreactor and cultured the scaffolds for 21 days with medium supplemented with GDF‐5 at 100 ng/mL. Immunohistochemistry, quantitative real‐rime polymerase chain reaction (qRT‐PCR), and western blot analyses were utilized to investigate stem cell commitment towards the tenogenic phenotype in the proposed environment.

## 2 MATERIALS AND METHODS

### 2.1 hTSPCs harvesting and culturing

Three healthy semitendinosus (males: 28, 43, and 51 years old) were obtained from non‐suitable tissue parts of semitendinosus autologous transplants after reconstruction of the anterior cruciate ligament, previous informed consent according to protocols approved by the Institutional Review Board of “San Giovanni di Dio e Ruggi D'Aragona Hospital” (Salerno, Italy) (Review Board prot./SCCE n. 151 achieved on October 29, 2020). The presence of comorbidities and any previous or concurrent tendon disease were considered exclusion criteria. hTSPCs were harvested from biopsies using a previously optimized method, cultured in minimum essential medium alpha (α‐MEM, Corning Cellgro, Manassas, VA, USA) supplemented with 1% Glutagro™, 1% penicillin/streptomycin (Thermo Fisher Scientific, Waltham, MA, USA), and 10% fetal bovine serum (FBS, Thermo Fisher Scientific, Waltham, MA, USA), and incubated at 37°C in an atmosphere of 5% CO_2_ and 95% relative humidity. Cells at passage 2 were used for immunophenotype characterization (Figure S2) and all further experiments.

### 2.2 HeLa cell culture

HeLa cells (CCL‐2™, ATCC) were cultured in Dulbecco's modified eagle medium (DMEM) supplemented with high glucose (4.5 g/L) (Thermo Fisher Scientific, Waltham, MA, USA) and supplemented with 10% fetal bovine serum (FBS, Thermo Fisher Scientific, Waltham, MA, USA) and 1% penicillin–streptomycin solution (Thermo Fisher Scientific, Waltham, MA, USA). Cells were maintained in a humidified incubator at 37°C with 5% CO_2_ atmosphere. The culture medium was replenished every 48–72 h, and cells were passaged upon reaching 70%–80% confluence using 0.25% trypsin–EDTA solution (Thermo Fisher Scientific, Waltham, MA, USA). Cells at passage 18 were used for the 3D bioprinted culture preparation.

### 2.3 ColMA scaffold and bioprinting process

Lyophilized PhotoCol® (Methacrylated Type I Bovine Collagen, Advanced Biomatrix, CA, USA) was resuspended upon arrival in 20 mM acetic acid at a concentration ranging from 4 to 8 mg/mL, following the manufacturer's protocol, and stored at 4°C. Lithium phenyl‐2,4,6‐trimethylbenzoylphosphinate (LAP, Advanced Biomatrix, CA, USA) was dissolved in complete α‐MEM (Corning Cellgro, Manassas, VA, USA) for hTSPCs, or DMEM (Thermo Fisher Scientific, Waltham, MA, USA) for HeLa cells at 17 mg/mL. A mixture of 32.9 μL of LAP, 146.7 μL of neutralization solution (provided in the PhotoCol® kit, Advanced Biomatrix, CA, USA), and 1.5 mL of PhotoCol® was prepared. Cells were then resuspended in the final master mix to obtain a final cell concentration of 1 × 10^6^ cells/mL of PhotoCol®, as previously optimized and described elsewhere.^28^, ^32^

Rokit Dr. INVIVO 4D bioprinter (RokitHealthcare, Seoul, Republic of Korea) was used to bioprint the final hydrogel mixture using a 22G nozzle and the syringe dispenser head. The printing bed temperature was set to 40°C, while the extrusion chamber temperature was set to 18°C. The printing speed of the extruder was set to 5 mm/s while the travel speed was set to 7 mm/s. The infill pattern selected was “concentric”, and the angle of rotation, and between each layer was set to 90°. Finally, the fill density was set to 25%. Via the NewCreatorK software (RokitHealthcare, Seoul, Republic of Korea, version 1.57.70), a concentric cylinder shape with a dimension of 5 mm diameter × 2 mm height, was created, sliced, and uploaded into the bioprinter. The scaffolds were printed on microscope glass coverslips and further crosslinked via UV radiation during bioprinting. The obtained scaffolds were then transferred to the perfusion bioreactor and cultured for up to 21 days.

### 2.4 Scaffold architecture characterization

The investigation into the shape and morphology of the bioplotted scaffolds was conducted using field emission‐scanning electron microscopy (FE‐SEM, model LEO 1525; Carl Zeiss SMT AG, Oberkochen, Germany).

For SEM observation, on day 0, 7, 14 and 21, samples were whashed with PBS, fixed with 4% paaformaldehyde, dehydrated by multiple passages across ethanol:water solutions (10 minutes each) with increasing percentages of ethanol and dried using a dense carbon dioxide at 200 bar and 38°C for 4 hours. Finally, were immersed in liquid nitrogen and fractured with a needle, before to be placed on a double‐sided adhesive carbon tape previously glued to an aluminium stub.

**FIGURE 1:**
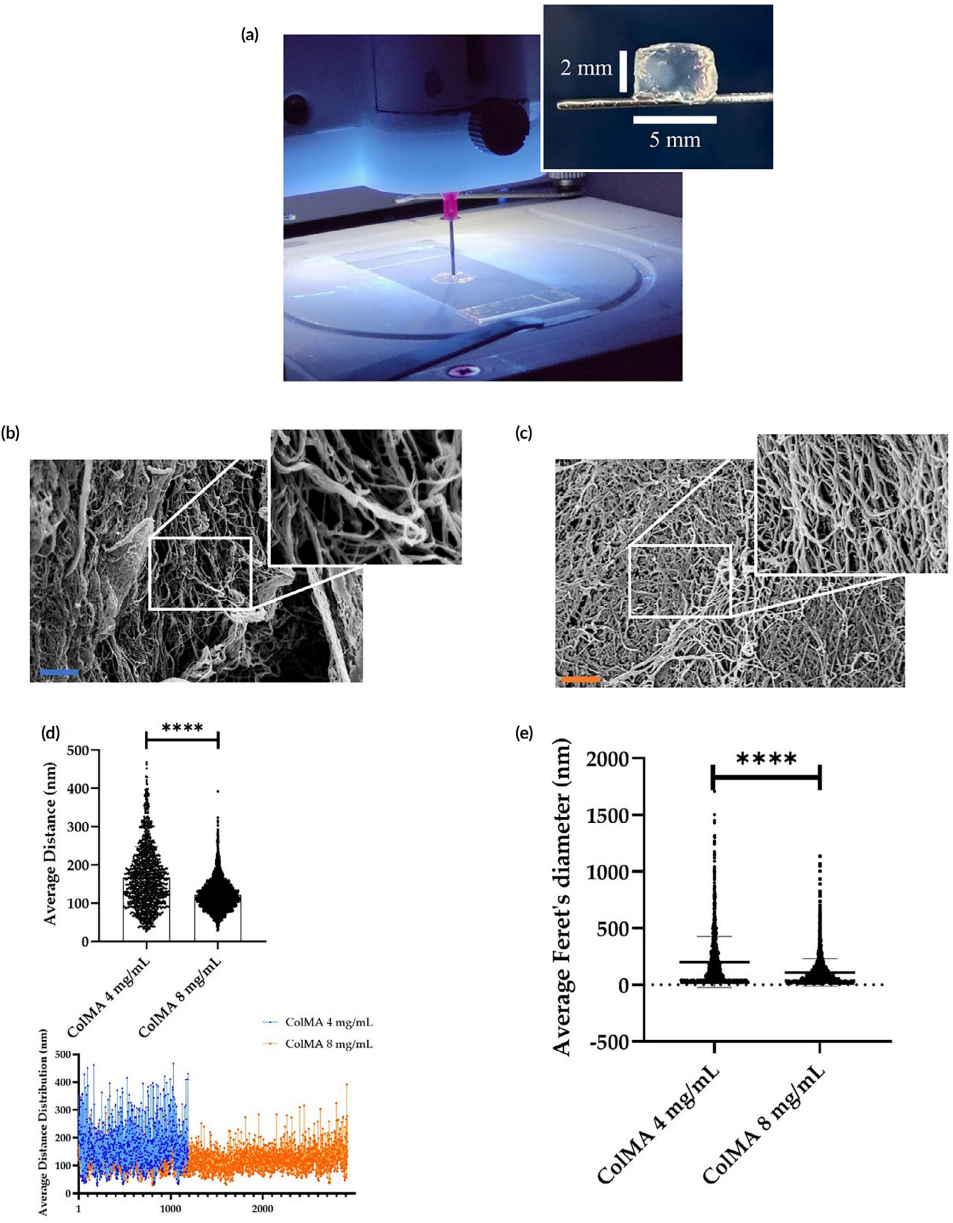
Advanced physicochemical characterization of empty ColMA bioprinted scaffolds at two different concentrations. Image of: the printing process with UV‐lights on and bioprinted scaffold after being cured and fully crosslinked (a); field‐emission scanning electron microscopy (FE‐SEM) at a concentration of 4 mg/mL (b) and 8 mg/mL (c) of PhotoCol®; scale bar is 3 μm; magnification: 20k×; average distance distribution analysis (d), and average Feret's diameter (e) of the bioprinted scaffold at both 4 and 8 mg/mL. Data were extrapolated using ImageJ software. ****p < 0.0001.

Before imaging, the samples were coated with a gold film (250&amp;#x02009;Å thickness) using a sputter coater (model 108 A; Agar Scientific, Stansted, UK). ImageJ (rel. 1.52p National Institutes of Health, Bethesda, MD, USA) software was used to automatically calculate Feret's diameter and distance of the four nearest pores. For the latter, the external NND plug‐in was used, following a described protocol.^33^, ^34^ The average Feret's diameter and the average distance distribution values were reported (Figure 1).

### 2.5 Dynamic culture

A custom perfusion bioreactor made of poly(methyl methacrylate) (PMMA, Altuglas® CN, La Garennecolombes, France), a biocompatible material for biomedical applications, was employed. The bioreactor consisted of a milled multi‐well plate with two holes allowing the insertion of silicon tubes (Tygon®, Charny, France) for continuous flow provided by peristaltic pumps at a constant rate of 1 mL/min. This continuous flow was maintained throughout the whole experiment. GDF‐5 was diluted in the cell culture media to obtain a tenogenic effect at a concentration of 100 ng/mL. The medium was recycled from the peristaltic pump and replaced once a week with a fresh one. The bioreactor system operated within a standard cell culture incubator (37°C with 5% of CO_2_).

### 2.6 Live/dead assay

Cell viability in the PhotoCol® bioprinted scaffolds with HeLa cells was detected by live/dead assay immediately after the bioprinting process, and on day 5 for HeLa cells, and on days 7, 14 and 21 for hTSPCs. Scaffolds were first washed three times in PBS 1× for 15 min, then immersed in the working solution composed of PBS 1× with 2% calcein AM solution (Cat. no. C1359, Sigma Aldrich, Milan, Italy), and 1% ethidium homodimer I solution (Cat. no. E1903, Sigma Aldrich, Milan, Italy), and placed in the incubator for 15 min. Lastly, scaffolds were washed again in PBS 1× for 5 min and imaged using a fluorescence microscope (mod. Eclipse, Nikon Corporation, Tokyo, Japan). Images were taken at 4× and 10× magnification. ImageJ (rel. 1.52p National Institutes of Health, Bethesda, MD, USA) software was used to measure the intensity of the signals. Original photos were taken in RGB format and then transformed into 16‐bit (gray‐scale) images. The tagged areas were then expressed as an average pixel intensity value between 0 (dark) and 255 (white).^35^

### 2.7 Hematoxylin and eosin staining

At the selected time points, scaffolds were fixed in 4% PFA for 2 h at RT, cryo‐protected in 30% sucrose (4 °C, overnight), included in the optimal cutting temperature (OCT) compound, and cut into slices of 15 μm thickness using a cryostat (CM 1950, Leica, Wetzlar, Germany). Scaffolds sections were subjected to gradual hydration keeping them at 54°C for 15 min, then at RT for 15 min, followed by a 5 min wash in MilliQ water. Following hydration, the samples were incubated with a hematoxylin and eosin staining solution for 60 min to visualize tissue. Subsequently, the sections were subjected to dehydration using an ascending ethanol gradient and then cleared in xylene for 5 min to remove excess staining and prepare them for imaging. The prepared sections were mounted onto slides using Eukitt (Sigma‐Aldrich) mounting medium to ensure optimal visualization and preservation of the tissue structure. Imaging of the stained sections was performed using an Olympus microscope BX53, equipped with a ProgRes SpeedXT core five camera.

### 2.8 Sirius red staining

Sirius red staining was conducted utilizing the Picrosirius Red Stain Kit (Polysciences, Inc., USA). Tissue sections, each measuring 15 μm in thickness, were initially stained with hematoxylin for 8 min, followed by a 2‐min wash in water. Subsequently, the sections were immersed in phosphomolybdic acid for 2 min, followed by another 2‐min water wash. Next, they were incubated in Picrosirius Red F3BA Stain for 60 min and then briefly dipped into a 0.1 M HCl solution for 2 min. To complete the staining process, the sections underwent dehydration in a series of ethanol gradients (70%, 75%, 95%, and 100%) before a final immersion in xylene for 5 min. Finally, the samples were mounted using Eukitt medium for subsequent analysis. Picrosirius red staining brightfield and polarized light images were captured using a Brunel polarization microscope equipped with a Nikon D500 camera using three different magnifications: 5×, 10× and 20×.

### 2.9 Immunofluorescence assay

Scaffold slices were permeabilized with 0.1% Triton X‐100 for 10 min and blocked with a 1% (w/v) solution of bovine serum albumin (BSA) for 1 h. For type I and type III collagen staining, slices were incubated with a mouse monoclonal anti‐type I collagen antibody (1:100, Sigma Aldrich, Milan, Italy) and a mouse monoclonal anti‐type III collagen antibody (1:50, Sigma Aldrich) overnight at 4°C. Subsequently, the slices were incubated at room temperature for 1 h with the DyLight 488 goat–anti‐mouse IgG (1:500, BioLegend, San Diego, CA, USA) and the Alexa Fluor™ plus 594 goat–anti‐mouse IgG (1:500; Thermo Fisher Scientific) antibodies, respectively. For tenomodulin and scleraxis staining, slices were incubated with a rabbit monoclonal anti‐tenomodulin antibody (1:100, Sigma Aldrich), and a rabbit monoclonal anti‐scleraxis antibody (1:100, Sigma Aldrich). The cell nuclei were counterstained with 4′,6‐diamidino‐2‐phenylindole (DAPI). Single images were captured using an inverted Leica laser‐scanning confocal microscope (mod. TCS SP5; Leica Microsystems, Wetzlar, Germany) equipped with a plan Apo 63X/1.4 NA oil immersion objective. The images were acquired with consistent light intensity, exposure time, and gain settings.

### 2.10 Gene expression and cytokine analysis

At each time point scaffolds were subjected to total RNA extraction using QIAzol® Lysis Reagent (Qiagen, Hilden, Germany) and the RNeasy Mini Kit (Qiagen, Hilden, Germany). Each sample utilized 1 μg of total RNA, which was reverse transcribed using the iScript™ cDNA synthesis kit (Bio‐Rad, Milan, Italy). Relative gene expression analysis was conducted on a LightCycler® 480 Instrument (Roche, Italy) employing the SsoAdvanced™ Universal SYBR® Green Supermix (Bio‐Rad, Foster City, CA, USA) and validated primers for SCX‐A, TNC, COL1A1, COL3A1, and TNMD (Bio‐Rad), following the MIQE guidelines. Triplicate experiments were performed for each studied condition, and the data was normalized to glyceraldehyde‐3‐phosphate dehydrogenase (GAPDH) expression, serving as the reference gene. The geNorm method^36^ was utilized to determine the stability of the reference gene across different conditions (calculated using CFX Manager software [v.3.1; Bio‐Rad, Milan, Italy]; *M* < 0.5). Fold changes were determined using the 2^−∆∆Ct^ method and presented as relative levels over day 0 (hTSPCs just after the bioprinting process). All experiments were performed in biological triplicates (*n* = 3).

Circulating cell culture media were thawed and analyzed with the human cytokine magnetic 10‐plex panel (Invitrogen, ThermoFisher Scientific), according to the manufacturer's instructions. This kit is designed for the quantitative determination of GM‐CSF, IFN‐γ, IL‐1β, IL‐2, IL‐4, IL‐5, IL‐6, IL‐8, IL‐10, and TNF‐α. The Luminex™ system uses microspheres marked with differing ratios of two different fluorophores, conjugated with monoclonal antibodies specific for different cytokines. In the assay, once the cytokine of interest has bound, a secondary detection antibody specific for the cytokine was added. The beads were read on a Luminex™ 100™. A serial dilution of the standards for the standard curve was added to the plate in duplicate. A positive sample was considered if it was above the limits of detection as determined by the manufacturer.

### 2.11 Western blot

For total protein extraction, at each time point, the scaffolds were subjected to a 15‐min wash in PBS, and placed into Buffer RLT (RNeasy® Micro Kit, QIAGEN, Germany). An equal volume of 70% ethanol was added to the lysate, followed by immediate centrifugation at 8000 *g* for 15 s. Subsequently, four volumes of ice‐cold acetone were introduced into the mixture and placed at −20°C for 30 min. The samples were then centrifuged at maximum speed for 10 min at 4°C and allowed to air‐dry. Finally, the samples were reconstituted in RIPA buffer (containing 150 mM NaCl, 1% Triton X‐100 at pH 8.0, 0.5% sodium deoxycholate, 0.1% SDS, and 50 mM Tris at pH 8.0) supplemented with protease and phosphatase inhibitors (Merck, USA). The protein concentration was determined using the Bradford assay (Bio‐Rad, Hercules, CA, USA), and 19 μg of total protein extracts were separated on SDS‐PAGE gels and subsequently transferred onto nitrocellulose membranes. The nitrocellulose blots were blocked in TBS‐T buffer (20 mM Tris–HCl, pH 7.4, 500 mM NaCl, and 0.1% Tween‐20) with 10% nonfat dry milk. They were then incubated overnight at 4°C in TBS‐T with 5% nonfat dry milk with the following primary antibodies: anti‐tenomodulin antibody (ab203676, Abcam, USA), anti‐β‐Tubulin antibody (F‐1, sc‐166729, La Santa Cruz Biotechnology, USA), and anti‐collagen type I antibody (ab138492, Abcam, USA). Immunoreactivity was detected by sequential incubation with appropriate horseradish peroxidase‐conjugated secondary antibodies (Merck) for 1 h at room temperature, followed by exposure to Pierce ECL detection reagents (Thermo Scientific, Rockford, IL, USA) for 1 min, using X‐ray film. Semi‐quantitative analysis of bands was conducted using ImageJ software (NIH, Bethesda, MD, USA; version 2.0.0‐rc‐54/1.51h). The relative gray area for each band was determined, and background values were subtracted from the calculations.

### 2.12 Statistical analysis

All experiments were performed at least three times, and the results were presented as mean values ± standard deviation (SD). Before statistical analysis, data were assessed for normality using Shapiro–Wilk's test, which is deemed suitable for small sample sizes (<50 samples). This test evaluates the null hypothesis assuming data are drawn from a population with a normal distribution. If the resulting *p*‐value exceeds 0.05, the null hypothesis is upheld, indicating normal data distribution. This prerequisite enables the application of parametric tests such as Student's *t*‐test and ANOVA. The normality test affirmed the normal distribution of the data, allowing for statistical analysis utilizing two‐tailed independent Student's *t*‐test for comparison between two independent groups or one‐way ANOVA, followed by Dunnett's multiple comparison test for differences among more than two independent groups. Significance was determined by *p*‐values <0.05. Statistical analyses were conducted using GraphPad Prism software (version 8.0 for Windows, LLC, San Diego, CA, USA), and findings were incorporated into the graphs along with legends.

## 3 RESULTS

### 3.1 Manufacturing protocol optimization and workflow standardization

In the optimization of the bioprinting protocol, various formulation and process parameters were examined. Collagen concentrations ranging from 4 to 8 mg/mL were tested. Table 1 summarizes the primary challenges encountered during the initial phase of optimizing these parameters, along with the solutions implemented. Key issues included moisture absorption by the matrix, hydrogel collapse at lower collagen concentrations, and crosslinking difficulties. To address these, the solutions involved diluting the sponge matrix, increasing the collagen concentration, and setting specific temperature parameters. These adjustments resulted in more homogeneous solutions, optimal collagen concentrations, improved printability, and successful crosslinking at the designated printing bed temperature. Furthermore, maintaining stable temperature conditions ensured better final results and experimental reproducibility, thus advancing scientific understanding and methodology in the field. With regard to other printing parameters, we initially focused on determining the optimal printing temperatures. Through experimentation, we established that the most effective conditions were 40°C for the printing bed and 18°C for the syringe cage.

**TABLE 1:** Main parameters optimized during ColMA's bioprinting.

Number	Parameter tested	Problem faced	Solution adopted	Outcome
1	Collagen concentration	4 mg/mL: collapsing hydrogel	Concentration increased up to 8 mg/mL	8 mg/mL as optimal working concentration
2	Workflow temperature	Crosslinking in the syringe	Low temperature (0–4°C) adopted during the whole protocol	Better printable master mix
3	Printing temperature	Crosslinking in the syringe	Syringe cage: 18°C Printing bed: 40°C	Solution was crosslinking at the right time

*Note*: The main issues faced during the optimization of the workflow to obtain a sustainable and reproducible protocol to bioprint PhotoCol® are summarized. The related solutions adopted are reported.

### 3.2 Physical characterization and software analysis of ColMA scaffolds

Advanced physicochemical characterization of the empty scaffolds was carried out using Fiel‐emission scanning electron microscopy (FE‐SEM) and digital analysis to provide information about their morphology and porosity. In more detail, Figure 1a displays the printing process and the final scaffold obtained after curing it in the cell incubator. Figure 1b,c display the FE‐SEM micrographs of the scaffolds obtained at 4 mg/mL and 8 mg/mL of ColMA concentration, respectively. It can be seen that there are several differences between the two concentrations as the average distance distribution plot highlights (Figure 1d, up): pores are higher in number (1915 pores vs. 2755 pores respectively), denser, and smaller in the 8 mg/mL compared to the 4 mg/mL, while the general structures of the filaments are comparable. There is less empty space in the higher concentration, giving it more niches to welcome cells, while remaining highly interconnected. Before incorporating cells, a more in‐depth analysis of the thickness of the wall and the average Feret's diameters was conducted through ImageJ software analysis. Specifically, the average distance between the four nearest neighbors was calculated (Figure 1d, low), and for the 4 mg/mL scaffold it averaged at 167 nm ± 80 nm; for the denser scaffold, it averaged at 122 nm ± 40 nm. The data collected between the two scaffolds were statistically different (Welch's test), confirming that the denser concentration is more packed with pores (which are also smaller when compared to the 4 mg/mL concentration), and those pores are closer to each other. Figure 1e depicts the average Feret's diameter distribution of pores on the scaffold surface. For the 4 mg/mL scaffold was 201 ± 225 nm; for the 8 mg/mL scaffold, the average Feret diameter was 110 ± 122 nm.

Cells vitality >90% was observed along plotting ColMa at 8 mg/mL in the case of HeLa line (Figure S1); therefore, this condition was adopted in the following.

### 3.3 Perfused dynamic culture helped nutrient exchanges during the cultivation of hTSPCs

**FIGURE 2:**
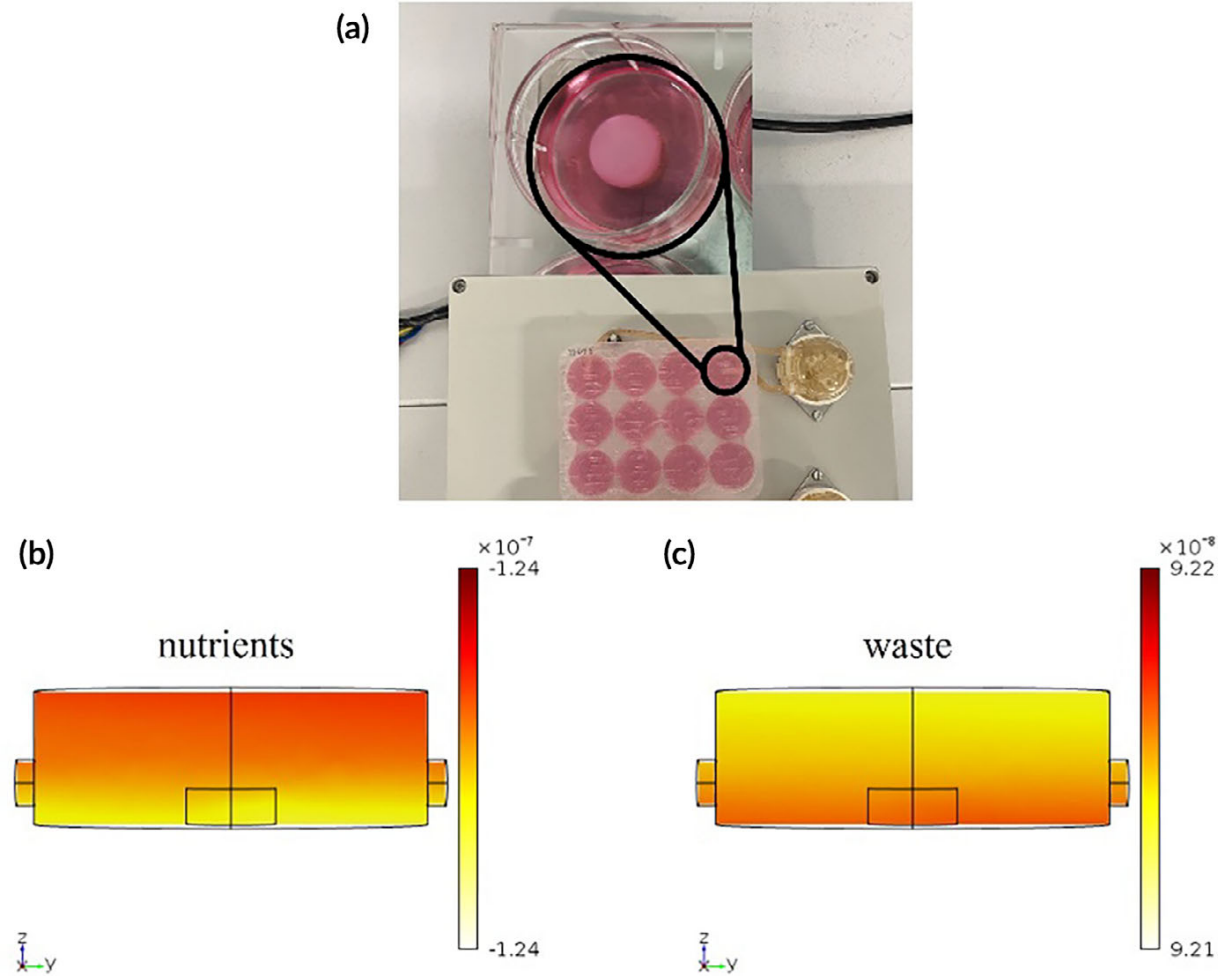
3D dynamic culture and FEM analysis. hTSPCs were cultured within the ColMA scaffold under dynamic conditions using a bioreactor. A perfusion bioreactor was employed, and the culture medium was supplemented with 100 ng/mL of hGDF‐5. Peristaltic pump settings included a flow rate of 1 mL/min (a). Finite element Modelling (FEM) analysis was utilized to assess nutrient (b), and waste concentration (mM/m^3^) within the scaffold‐media interface (c).

hTSPCs were isolated following an established protocol^4^ and integrated at a concentration of 1 × 10^6^ cells/mL. The bioprinting process was successfully executed, with cell viability exceeding 90% after processing. The successful bioplotting of primary cells to fabricate a complex 3D in vitro model validated our workflow protocol. The 3D scaffolds were cultured under dynamic conditions, with the medium supplemented with 100 ng/mL of human growth/differentiation factor‐5 (hGDF‐5) to induce tenogenic differentiation.^23^, ^28^ A flow rate of 1 mL/min was administered to assure continuous perfusion within the cell culture environment.^32^ A finite element modeling (FEM) approach was employed to assess nutrient and waste product exchanges during the cell culture period. The model incorporated considerations of laminar flow and mass transport to accurately simulate the microenvironment conditions within the scaffold,^32^ with a permeability coefficient of hydrogel assumed as 2 × 10^−9^ cm^2^,^37^ assumed as boundary conditions. Figure 2 shows that the dynamic environment results in a uniform distribution of both nutrients and waste products within the single culture well and across the 3D construct.

### 3.4 Live/dead assay with primary cells showed good viability

**FIGURE 3:**
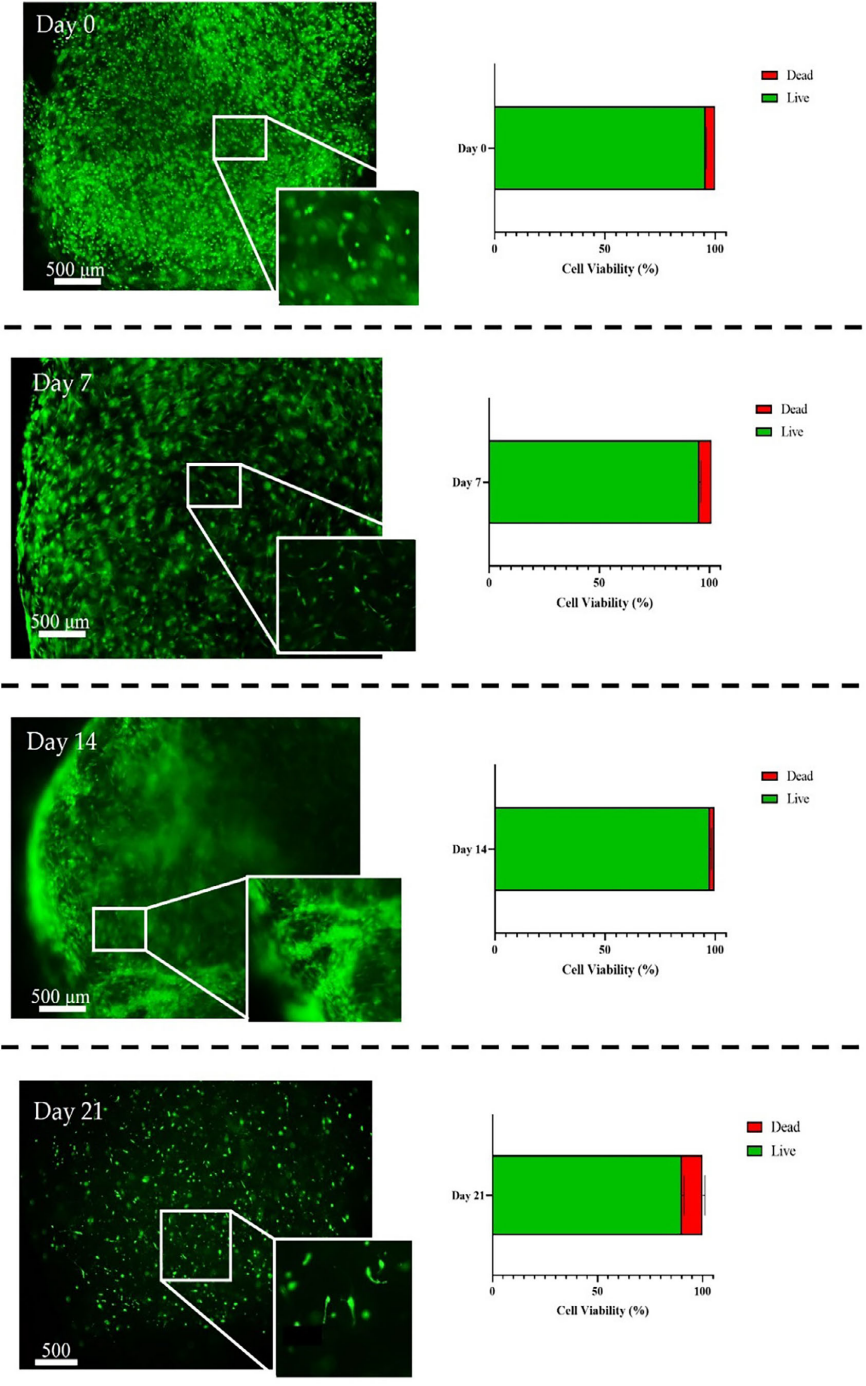
Live/dead assay on hTSPCs bioprinted scaffolds. hTSPCs were used to bioengineer 3D ColMA scaffolds and cultured under perfusion for 21 days in a GDF‐5‐supplemented medium. Live/dead assay was performed at every culture time‐point (0, 7, 14, and 21 days) and images were acquired at both 4× and 10× magnification; live cells are stained in green while dead cells are stained in red. *N* = 3 (biological replicates). Scale bar: 500 μm.

Live/dead assay of hTSPCs at various time points along dynamic culture, including 0, 7, 14, and 21 days was illustrated in Figure 3, with semi‐quantitative analysis of the live/dead signals. Living cells are stained in green, while those non‐viable in red. The data documented highly cell viability just after the printing process (approximately 90% viability) and also throughout the entire duration of dynamic culture, where viability consistently exceeded 85%. Interestingly, as observed in the zoomed micrographs, by day 21, cells exhibited an elongated morphology, resembling mature tenocytes.^23^ It has to be added that visible scaffold total volume reduction of about 40% was also observed along the culture, suggesting cells interaction with external collagen fibers belonging to the synthetic scaffold.

### 3.5 Sirius red staining reveals collagen matrix's remodeling

**FIGURE 4:**
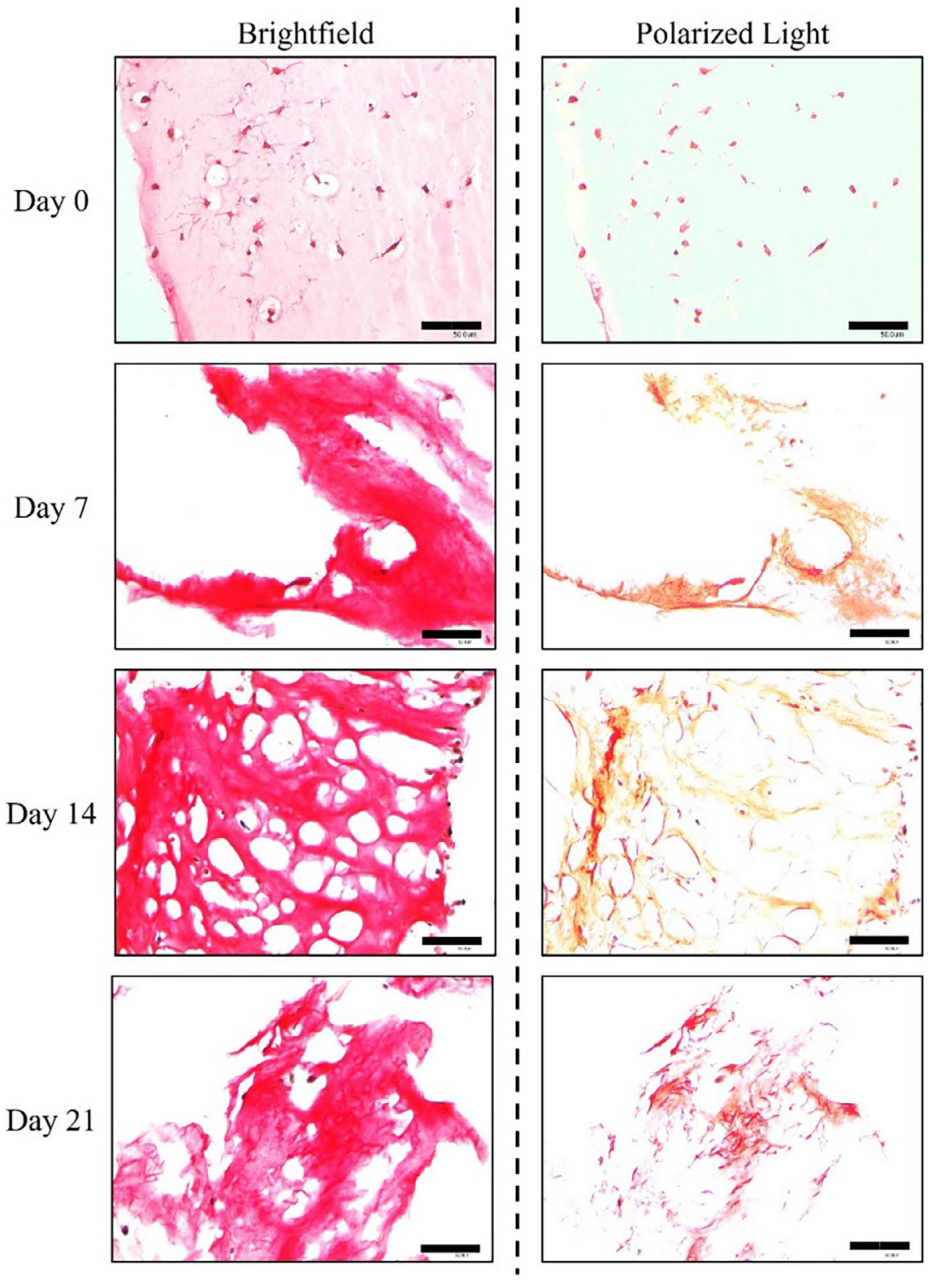
Sirius red staining of hTSPCs cultured in ColMA scaffolds for up to 21 days under dynamic conditions with GDF‐5 supplementation. Sirius red staining at different time points was performed to assess type I collagen (in red, visible under normal light, and red‐yellow or orange under polarized light) and type III collagen (in green under polarized light) production within the PhotoCol® scaffold. Total collagen is visible in red under normal light (left panel), while cross‐polarized light marks type I collagen in bright yellow‐orange and type III collagen in green (right panel). *N* = 3 (biological replicates). Scale bar: 500 μm.

Sirius red staining revealed significant production of type I collagen, which appeared red under normal light and bright yellow or orange under polarized light (see Figure 4). Additionally, type III collagen, which should appear green under polarized light, was not detected in the 3D matrix. Over the 21‐day culture period, a notable transformation in the architecture and structure of the PhotoCol® scaffold was observed. Starting from day 7 (with the scaffold initially exhibiting almost complete whiteness or pink coloration on day 0), cells began to produce new collagen, a process that continued through to the day 21. Type III collagen was not detected within the 3D matrix.

### 3.6 Type III collagen showed up‐regulation in qRT‐PCR assay

**FIGURE 5:**
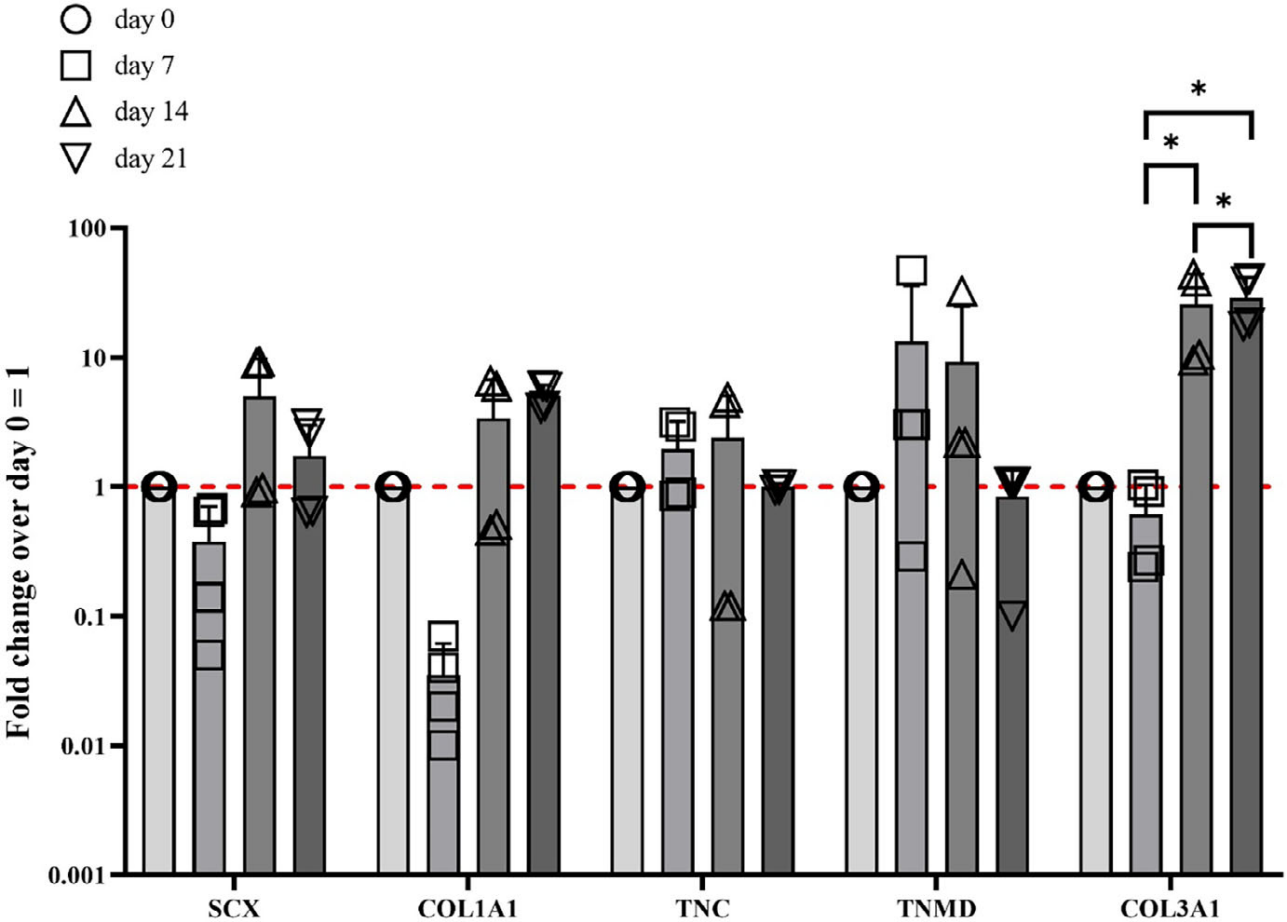
mRNA expression levels of the main tenogenic markers by hTSPCs cultured in 3D bioprinted scaffolds under dynamic conditions, and in the presence of GDF‐5. hTSPCs were cultured within ColMA scaffolds for up to 21 days. The expression of different tenogenic markers (SCX, COL1A1, TNC, TNMD, COL3A1) was investigated by qRT‐PCR. Data were normalized to GAPDH expression and presented as fold change over day 0 (hTSPCs just after the bioprinting process). The Y‐axis is in logarithmic scale. *N* = 3 (biological replicates); *p < 0.05.

qRT‐PCR was performed on scaffold samples collected on days 0, 7, 14, and 21 to investigate the mRNA levels of specific tenogenic markers, including SCX‐A, COL1A1, TNC, TNMD, and COL3A1 (Figure 5). Results indicate that by day 21, SCX (1.73 fold), TNMD (0.84 fold) COL1A1 (5.05 fold), and COL3A1 exhibited upregulation. However, among them, only COL3A1 displayed statistically significant upregulation (40‐fold, *p* < 0.05) on days 14 and 21.

### 3.7 Evaluation of GDF‐5 supplementation on structural protein production

**FIGURE 6:**
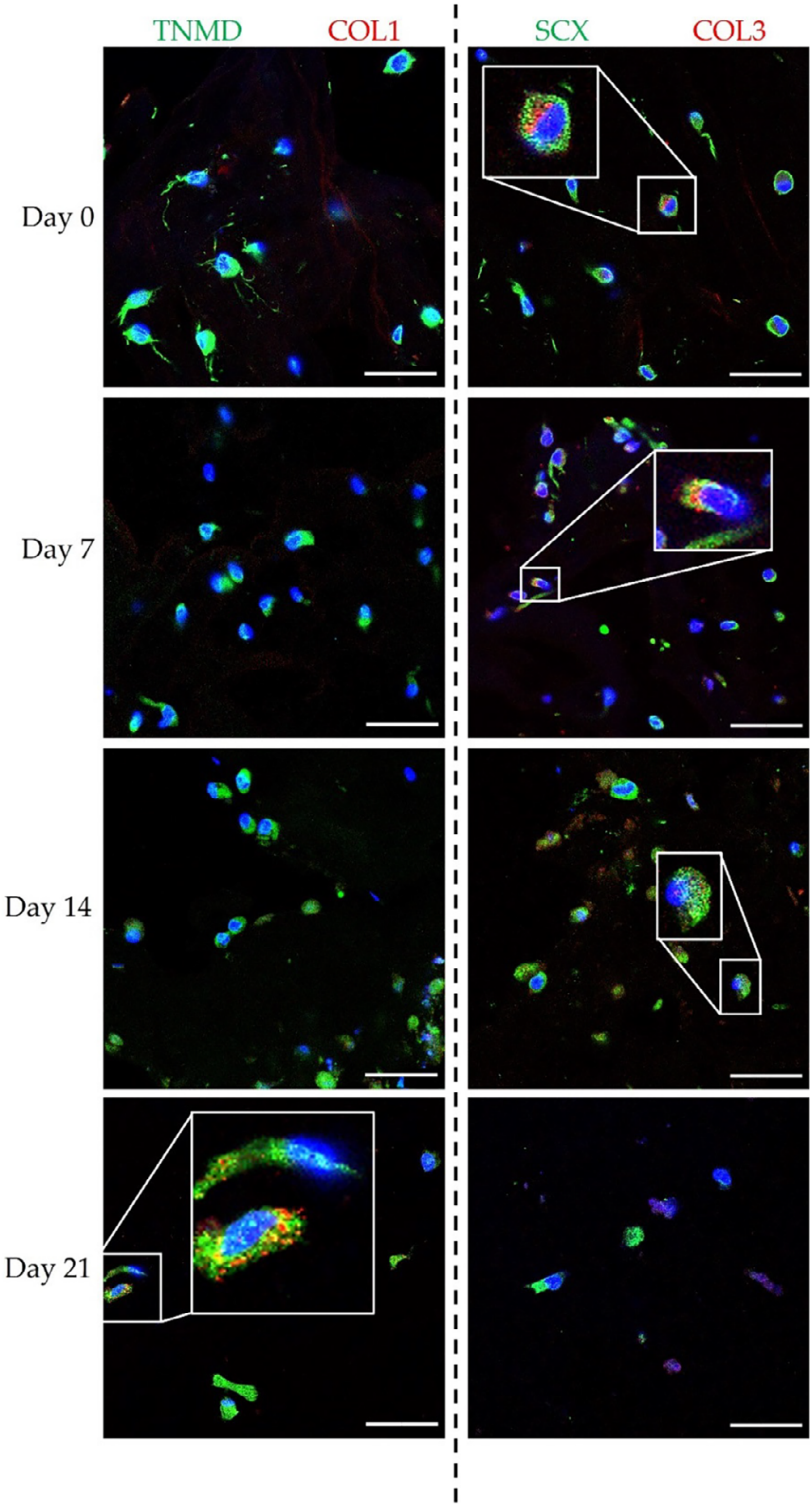
Immunofluorescence analysis of hTSPCs‐bioengineered ColMA scaffolds up to 21 days. Representative immunofluorescence images of hTSPCs cultured under dynamic conditions and treated with GDF‐5. Tenomodulin is labeled in green, while type I collagen is labeled in red (left panel). In the right panel, type III collagen is shown in red, and scleraxis is shown in green. Nuclei are stained in blue. *N* = 3 (biological replicates). Scale bar: 50 μm.

Immunofluorescence assays were performed to assess the expression of tenomodulin, type I collagen, scleraxis, and type III collagen within the 3D ColMa scaffolds at each time point, with representative images presented in Figure 6. On day 21, the presence of collagen type I was clearly observed, while tenomodulin remained consistently visible throughout the experiment. Scleraxis showed prominent expression at all time points. Collagen type III was detected on days 0 and 7, but its intensity decreased by days 14 and 21.

### 3.8 Evaluation of GDF‐5 supplementation of circulating proteins

**FIGURE 7:**
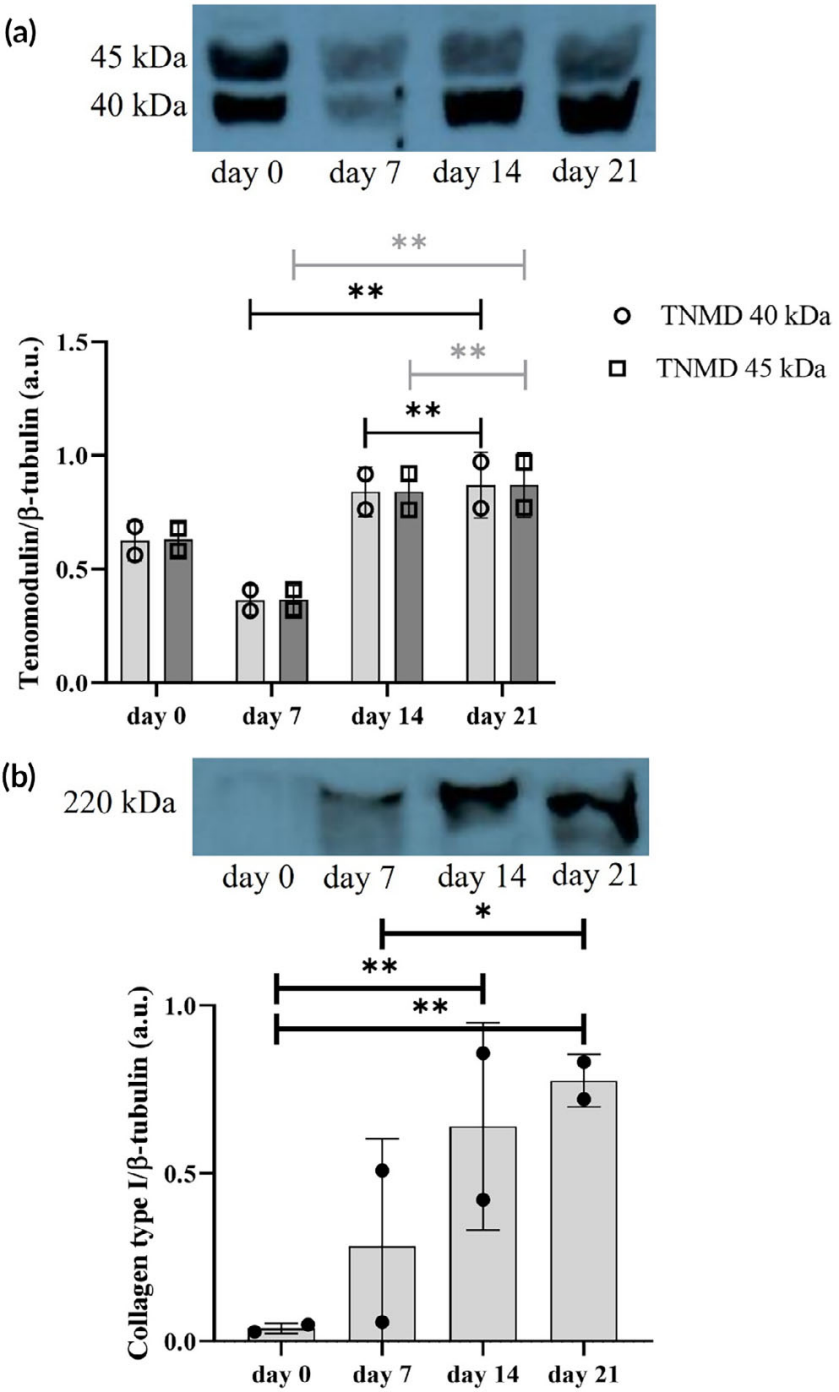
Western Blot and related semi‐quantitative analysis of proteins extracted from 3D bioprinted scaffolds bioengineered with hTSPCs under dynamic culture conditions and GDF‐5 supplementation. Glycosylated (45 kDa), and non‐glycosylated (40 kDa), forms of tenomodulin (a) and type I collagen (b) were measured in hTSPCs on days 0, 7, 14, and 21. Notably, tenomodulin exhibits a statistically significant upregulation at day 7 compared to days 14 and 21, while type I collagen displays a progressive over‐expression trend from day 0 to day 21. Data are shown as the mean ± SD of *N* = 2 independent experiments (biological replicates). *p < 0.05, **p < 0.01.

Western blot analysis was performed on proteins extracted from cells seeded within collagen 3D scaffolds (Figure 7). A statistically significant difference in tenomodulin expression between day 21 compared to both day 7 and day 14. This statistically significance difference is reported for both forms: glycosylated (45 kDa), and non‐glycosylated (40 kDa) tenomodulin.^38^ For type I collagen, a statistically significant difference was observed between day 0 and day 21, as well as between day 0 and day 14, with a *p*‐value of 0.01. Additionally, a significant difference was noted between day 7 and day 21, with a *p*‐value of 0.05. These results indicate a clear trend of progressive over‐expression of type I collagen, reflecting the proper progression of cell differentiation towards a tenogenic phenotype.

### 3.9 Evaluation of circulating cytokines in the culture media

**FIGURE 8:**
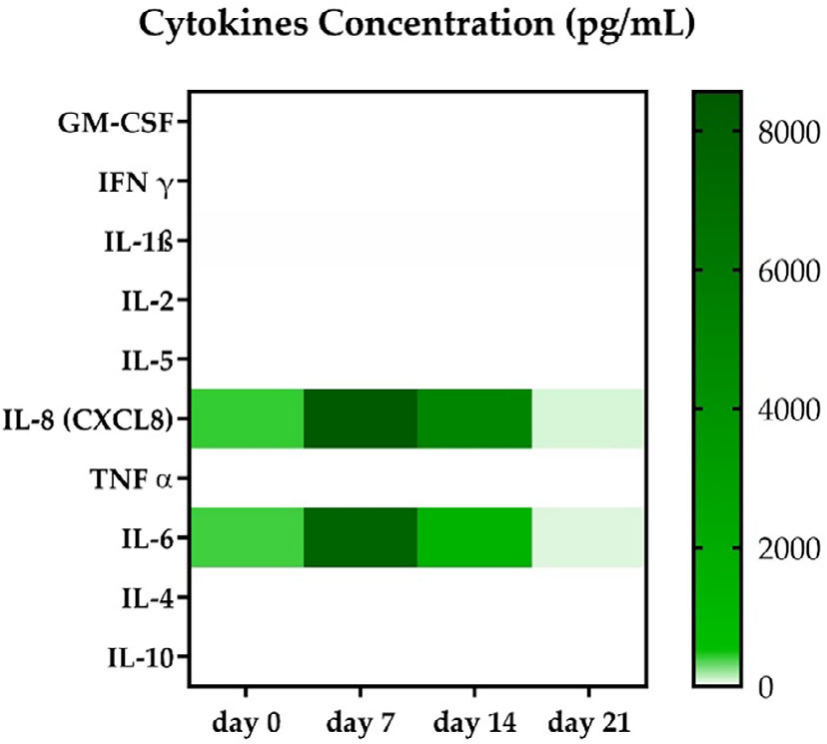
Heat map of 10 cytokines concentration extracted from the culture media of 3D scaffolds bioengineered with hTSPCs, cultured in a perfusion bioreactor, supplemented with GDF‐5. Circulating levels of interleukin 8 and interleukin 6, while initially present, spiked on day 7 and then gradually decreased over day 14 and day 21. Among the other tested cytokines, only interleukin 1β was found in traces (<0.1 pg/mL), thus not graphically appearing in the image.

Cell culture media was collected at each time point and tested for cytokines detection (Figure 8). The findings revealed the presence of interleukin 8 (IL‐8) and interleukin 6 (IL‐6) at day 0, with concentrations of 278 and 263 pg/mL, respectively. On day 7 both pinned at 8566 and 7636 pg/mL, to then start to decrease from day 14 ahead. At day 21 IL‐8 was found to be at 54 pg/mL while IL‐6 was found to be at 46 pg/mL. Of all the other cytokines tested, only interleukin 1β (IL‐1β) was also detected, but there were traces of it at all time points (<0.1 pg/mL).

## 4 DISCUSSION

To mitigate one of the primary drawbacks associated with natural collagen polymers, such as batch‐to‐batch variations, commercially available methacrylated collagen (PhotoCol®) has been utilized, which is compatible with cell incorporation and manual extrusion, but was not listed for bioprinting by the manufacturer (Advanced Biomatrix).

So, the primary objectives of this study were to establish a consistent workflow and printing protocol for the commercial PhotoCol® bioink using bioprinting technology. Indeed, ColMA managing during the bioplotting procedure is quite difficult and often unsuccessful due to the lack of precise control of the hydrogel viscosity (due to its peculiar thermal inertia); it happens both when loading printer syringe and along plotting. This issue highly reduced the printing resolution and proper scaffold final shape. Furthermore, this lack of proper hydrogel viscosity control lowered its application in biofabricating 3D in‐vitro models with primary cells such hTSPCs, which are extremely sensitive to the extrusion step.

To better manage ColMa viscosity issue, different collagen concentrations ranging from 4 to 8 mg/mL were tested, and the best printability was observed at 8 mg/mL; in this condition, we obtained a proper crosslinking with good printing resolution. Probably, this last operative concentration facilitated the increase of thermic inertia of the denser mixture, which needed to be maintained all at 10°C, avoiding any temperature profile. Furthermore, adopting at least 1.5 mL of the final mixture for each run, further enhanced the thermic inertia of the system, making it easier to maintain the temperature of the mixture below 10°C, along the printing workflow. On the other hand, the syringe cage was set to 18°C to gradually heat the PhotoCol®, while complete thermic crosslinking was achieved by setting the printing bed at 40°C. These adjustments resulted in a robust and reproducible working protocol for extruding PhotoCol®, allowing progression to the next stage.

Then, the subsequent focus was the optimization of the printing speed and nozzle size.^39^ Experiments were conducted with printing speeds ranging from 5 to 10 mm/s and nozzle sizes from 27 to 22 G. A printing speed of 5 mm/s was selected as the best, using a nozzle size of 22 G (0.70 mm internal diameter); indeed in these conditions, it was never observed any needle blockages coupled with excellent cell vitality, suggesting that probably the shear stress during the hydrogel extrusion is minimized. Indeed, a preliminary study by live/dead assay on cell viability with HeLa line indicated viabilities between 90% and 95% of plotted cells when their concentration was maintained at 1 × 10^6^ cells/mL of PhotoCol®^28^, ^32^, ^40^ (Figure S1).

Morphological characterization of the collagen scaffold by FE‐SEM analysis indicated that the scaffold printed at a concentration of 8 mg/mL had more pores (even if smaller in mean size) than the one obtained at a lower concentration of 4 mg/mL (i.e., 2755 vs. 1193 casualties, respectively). The mean distance among the four nearest neighbors was also smaller in the higher concentration (Figure 1). Due to the better printability behavior and considering that the void spaces were still suitable for cell culture, 8 mg/mL was chosen as the best concentration for hTSPCs bioplotting.

hTSPCs were biofabricated in the same 3D environment and their concentration was maintained at 1 × 10^6^ cells/mL. This value was chosen based on previous study with different stem cells even if in a different hydrogel 3D environment.^24^, ^28^ Under these conditions, hTSPCs were successfully bioplotted with a viability of 96% and were subsequently cultured for 21 days using a perfusion bioreactor to ensure adequate mass transport of nutrients and waste (Figure 2). Cell viability remained consistently above 85% throughout the 21‐day culture period, under perfusion. We fabricated a cylindrical scaffold (5 mm in diameter; 2 mm in height) because its simple geometry, defined by radius and height, makes it easier to model. The uniform properties along the height of the scaffold simplify the analysis of flow patterns. Additionally, the well‐established equations governing cylindrical coordinates and the application of rotational symmetry help reduce computational complexity by allowing us to model only a portion of the scaffold and then extrapolate the results to the entire structure. Our mass transfer FEM indicated that the dynamic environment results in a uniform distribution of both nutrients and waste products within the single culture well and across the 3D construct, at the flow rate selected of 1 mL/min.

The medium was supplemented with 100 ng/mL of hGDF‐5 to promote tenogenic differentiation.^23^ This concentration was selected based on prior studies showing its effectiveness in enhancing tenogenic differentiation of stem cells without adverse effects. Specifically, 100 ng/mL of GDF‐5 has been demonstrated to significantly increase the expression of tenogenic markers in both hBM‐MSCs and those derived from hWJ‐MSCs.^23^ Elongated‐shaped cells were observed within the 3D scaffold on day 21, indicating a positive evolution of the cell phenotype (Figure 3).

hTSPCs behavior along the culture was further characterized by histology assay with Sirius red. Optical microscope observation showed clearly a structural and architectural remodeling of the collagen within the 3D scaffold slices (Figure 4). Indeed, from day 7 new collagen fibrils are evident under polarized light, reasonably produced by cells, given the fact that at day 0 almost no collagen is displayed, under both normal and polarized light. In addition, mainly type I collagen was detected, whereas, the absence of type III collagen under polarized light has to be underlined. Collagen type I is the preferable type of collagen because it is associated with a higher quality given that its tendon fibrils are wider and more structurally organized than type III collagen. It is also possible to distinguish the collagen synthesized by the cells from the collagen of the scaffold because it was used a collagen type I antibody that specifically binds to human collagen, distinguishing it from the bovine‐derived collagen in the hydrogel.

The qRT‐PCR analysis revealed distinct patterns of gene expression, particularly notable for tenomodulin, which showed an upregulation at day 7 followed by a decrease at days 14 and 21. This fluctuation in gene expression, particularly for tenomodulin, may reflect dynamic processes within the scaffold and temporal shifts in cellular differentiation or response to environmental cues. The expression of the type I collagen gene also exhibited interesting trends, showing an upregulation at day 14, coinciding with the upregulation of other tendon‐related genes (see Figure 5). On the other hand, type III collagen is usually associated with injiuries and scar tissue whiel an increasing of type III/type I collagen is found higer in older subjiects.^41^ Type III collagen showed a statistically significant difference at day 14 and 21 when compared to day 7, but also between day 14 and day 21. However, the immunofluorescence analysis did not conclusively confirm the presence of these collagen type in the scaffold, with type III collagen observed only at earlier time points and type I collagen detected only at day 21 (Figure 6). Interestingly, Western blot analysis of tenomodulin production revealed a different temporal pattern compared to qRT‐PCR, with a slight decrease at day 7 followed by an increase at days 14 and 21 (Figure 7). This discrepancy could underscore the complexity of cellular responses within the scaffold. In addition to the gene expression and protein analysis, the investigation of the cell culture media supplemented with GDF‐5 provided further insights into the cellular response within the collagen matrix (Figure 8). Traces of IL‐1β were found at all time points while a very defined pattern could be drawn for IL‐8 and IL‐6. They were detected at day 0, spiked at day 7, and then gradually vanished at day 21 where their concentrations were found to be 54 and 46 pg/mL respectively. IL‐6 was found to actively being involved in the development of tendon disease and tendon tears, alongside an array of other cytokines.^42^ IL‐8 on the other hand, was found to not only be an active part of ECM remodeling, biomechanical adaptiveness, and tissue homeostasis,^43^ but also be a key factor in some chronic diseases such as rheumatoid arthritis. In more detail, it was found to play an essential role in various tissues in animal models of arthritis.^44^

The observed steady decrease in IL‐6 and IL‐8 concentrations, following an initial spike on day 7, suggests a mitigation of their inflammatory role in our model. This pattern may reflect a dynamic interplay between GDF‐5 supplementation and the secretion of pro‐inflammatory cytokines, potentially influencing cellular behavior and tissue remodeling processes within the engineered scaffold. The temporal dynamics of cytokine secretion, in conjunction with gene expression and protein production, underscore the complex regulatory mechanisms involved in tissue development and response to growth factor stimulation. These findings highlight the need for further investigation into the functional implications of cytokine dynamics for tissue engineering applications. Conversely, our 3D environment appeared to be highly promising for further studying tenogenic events.

## 5 CONCLUSIONS

This study introduces a novel and robust pipeline for bioprinting ColMa hydrogels with primary cells. PhotoCol® bioink was successfully developed, showcasing its potential for creating 3D in vitro models to study tenogenic processes using hTSPCs. The research provides valuable insights into the behavior of hTSPCs within the printed scaffolds and their response to biochemical stimuli, offering a foundation for future tissue engineering and regenerative medicine research. Looking ahead, this work opens the door to exploring 3D cultures constructed with pathological hTSPCs, which could offer critical insights into the biochemical alterations associated with tendon pathologies. Additionally, this approach may facilitate the investigation of methods to restore impaired biological functions. Future directions may include the incorporation of nanoparticles and nanomedicine formulations into the 3D printed environment, aiming to influence cell differentiation or investigate cellular responses.

## AUTHOR CONTRIBUTIONS

**Giacomo Cortella:** Methodology; writing – original draft; investigation; data curation. **Erwin Pavel Lamparelli:** Methodology; writing – review and editing; investigation; formal analysis; software. **Maria Camilla Ciardulli:** Methodology; writing – review and editing; investigation; formal analysis; software. **Joseph Lovecchio:** Software; data curation; writing – review and editing. **Emanuele Domenico Giordano:** Validation; supervision; writing – review and editing. **Nicola Maffulli:** Supervision; funding acquisition; writing – review and editing. **Giovanna Della Porta:** Conceptualization; data curation; validation; formal analysis; supervision; project administration; writing – review and editing; funding acquisition.

## FUNDING INFORMATION

This project was partially supported from the European Union's Horizon 2020 research and 753 innovation program under the Marie Skłodowska‐Curie grant agreement no. 955685, 754 www.helsinki.fi/p4fit. In detail, hTPSCs extraction and harvesting protocol was implemented and optimized along the P4FITproject. This research also received funding support from FARB UNISA 2021 and FARB UNISA 2022 Prof. Della Porta.

## CONFLICT OF INTEREST STATEMENT

The authors declare no conflict of interest. The funders had no role in the design of the study; in the collection, analyses, or interpretation of data; in the writing of the manuscript, or in the decision to publish the results.

### PEER REVIEW

The peer review history for this article is available at https://www.webofscience.com/api/gateway/wos/peer-review/10.1002/btm2.10723.

## Supporting information


**Appendix S1:** Supplementary materials.

## Data Availability

The data that support the findings of this study are available from the corresponding author upon reasonable request.
