# Supplementary material for: ColMA‐based bioprinted 3D scaffold allowed to study tenogenic events in human tendon stem cells
Source: Bioeng Transl Med. 2024 Oct 30;10(1):e10723. doi: 10.1002/btm2.10723 (PMC11711214; doi:10.1002/btm2.10723)
Supplement: Supplementary file 1 — Appendix S1: Supplementary materials. [file BTM2-10-e10723-s001.docx]

**SUPPLEMENTARY MATERIALS:**

1. Cell Viability after biofabrication process

Once established that a concentration of 8 mg/mL was the most suitable for our needs, we proceeded to assess the cytocompatibility of this process using a well-established cell model, HeLa cells. Cells were resuspended in the cell culture media at a concentration of 1x10^6^ cells/mL, a concentration reported as optimal in prior works. The Live/Dead indicated that HeLa cells exhibited high viability immediately after the printing process, with over 85% of the cells being alive. This high cell viability persisted for up to 5 days, demonstrating the effectiveness of our process in maintaining cellular health and functionality (**Figure S1**).

1. **Flow Cytometry and Gating Strategy of hTSPCs**

hTSPCs (passage 2) were detached and counted, and a total of 1x10^5^ cells were subjected to incubation at room temperature (RT) for 20 minutes with directly conjugated mouse–anti-human antibodies, including CD34-PE, CD90-FITC, CD105-PE, HLA class-II-FITC, and CD14-PC7 (all sourced from Beckman Coulter, Fullerton, CA, USA), as well as CD73-APC (Miltenyi Biotec, Gladbach, Germany). Following antibody incubation, the samples underwent two washes with PBS 1x (Corning Cellgro) and were then resuspended in the same buffer for subsequent analysis. Sample acquisition was carried out using a BD FACSVerse flow cytometer (Becton Dickinson, BD, Franklin Lakes, NJ, USA), equipped with two lasers (blue: 488 nm, and red: 628 nm). Compensation adjustments were determined utilizing single-color controls for each fluorochrome, along with an unstained sample serving as a negative control for setting PMT (photomultiplier tube) voltages. Consistent PMT voltages were applied across all samples. A minimum of 30,000 events were recorded during acquisition. FlowJo software (version 10.7.1, LLC, BD Biosciences, Franklin Lakes, NJ, USA) was utilized for post-acquisition compensation and subsequent flow cytometric analysis. Identification of hTSPCs was initially performed based on linear parameters (forward scatter area [FSC-A] vs. side scatter area [SSC-A]), with doublet exclusion accomplished via FSC-A vs. FSC-H gating. Expression levels of each marker on single cells were assessed through histogram analysis, utilizing an unstained sample as a reference for negative control determination (**Figure S2**).

1. **Western Blotting:**

As requested during revision, here are attached the images of the Western Blotting investigation of Type I Collagen (**Figure S3**), and Tenomodulin proteins (**Figure S4**).


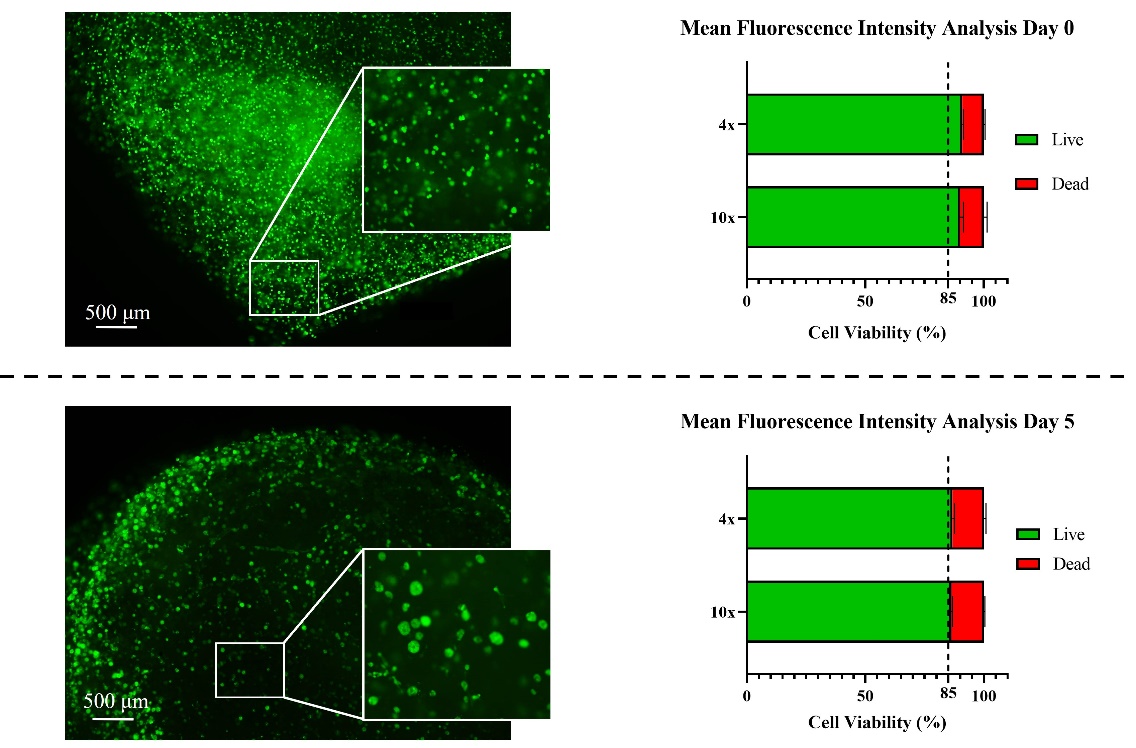


**Figure S1: Live/Dead assay and semi-quantitative analysis of cell viability.**

Live/Dead assay of HeLa cells right after the bioprinting process **(up)**, and after 5 days of static culture **(down)** (magnification 10X). Living cells are stained in green while dead cells are stained in red. Signal intensity in each time point was quantified by ImageJ software and data are shown as mean ± SD. Scale bar: 500 μm.


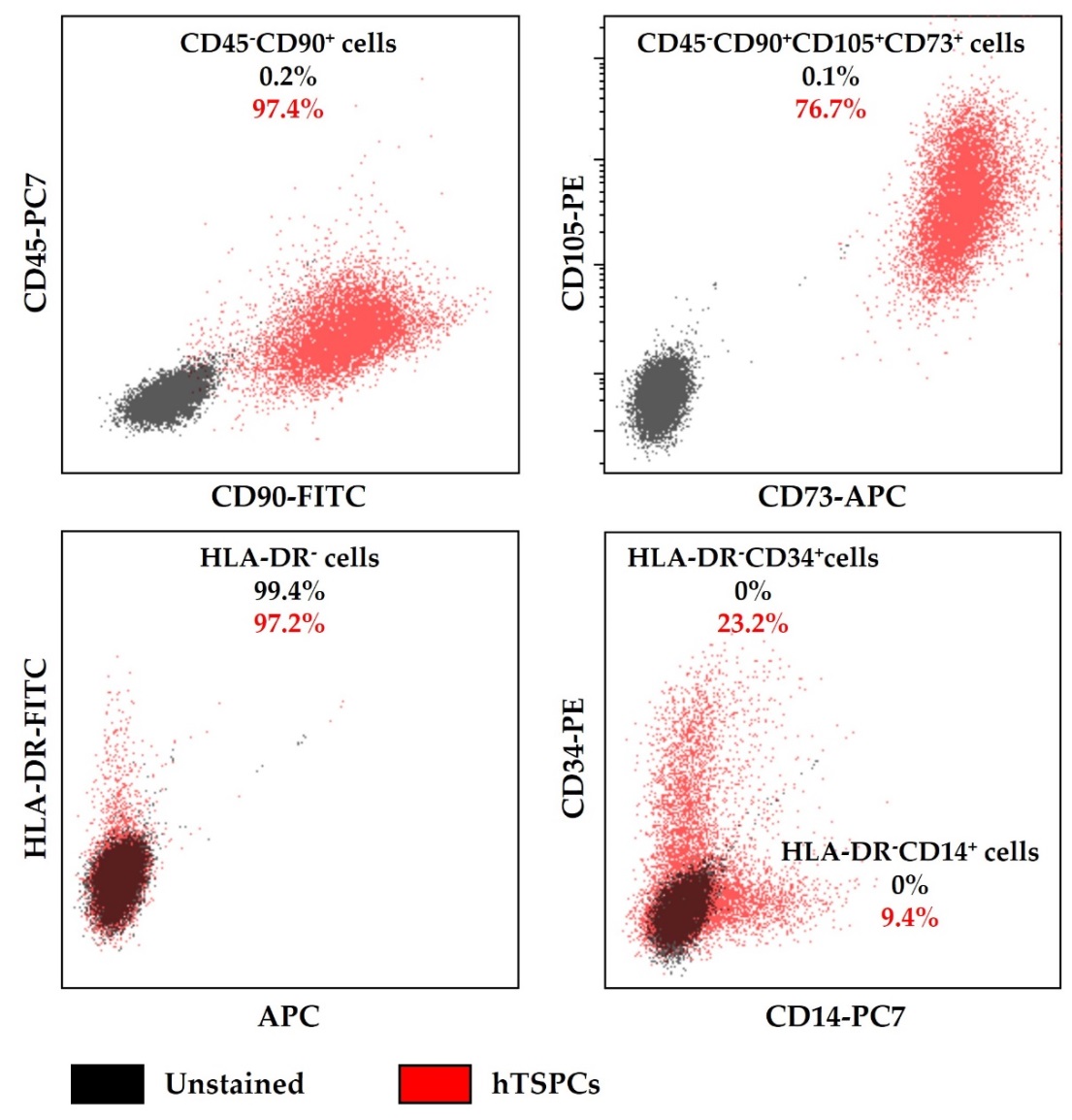


**Figure S2: Flow cytometry analysis of hTSPCs isolated from healthy tendon samples.**

The panel illustrates surface marker expression of hTSPCs, including CD45, CD90, CD73, CD105, HLA-DR, CD34, and CD14. The flow cytometry analysis revealed that hTSPCs exhibited positive expression of mesenchymal stem cell surface markers (CD90, CD73) and CD105. However, they were negative for CD45, HLA-DR, CD34, and CD14. N=3 (biological replicates).


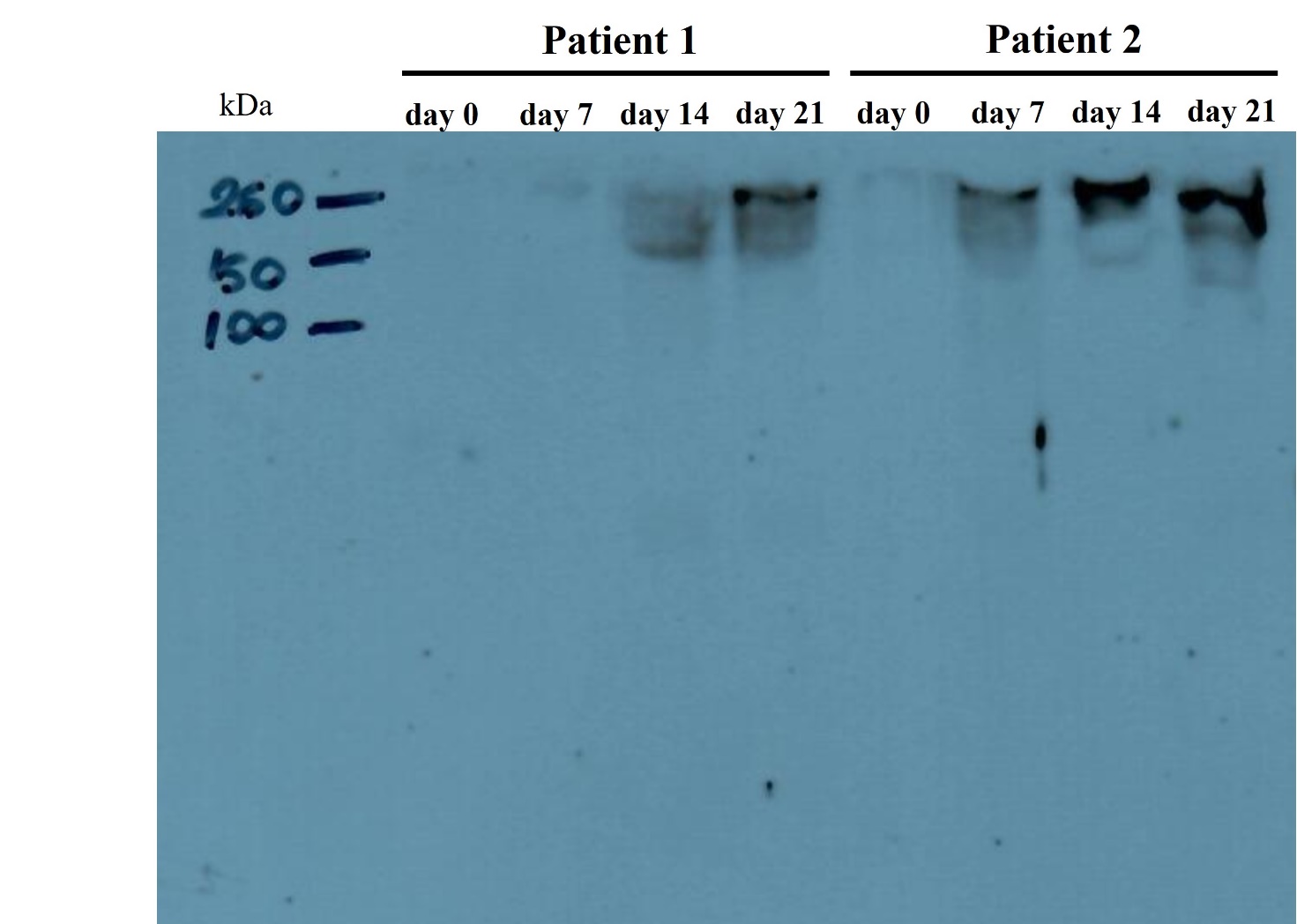


**Figure S3: Western blot image investigating Type I Collagen expression.**

Expression of the Type I Collagen protein extracted from the functionalized scaffold after 0,7,14, and 21 days of culture. N = 2 biological replicates.


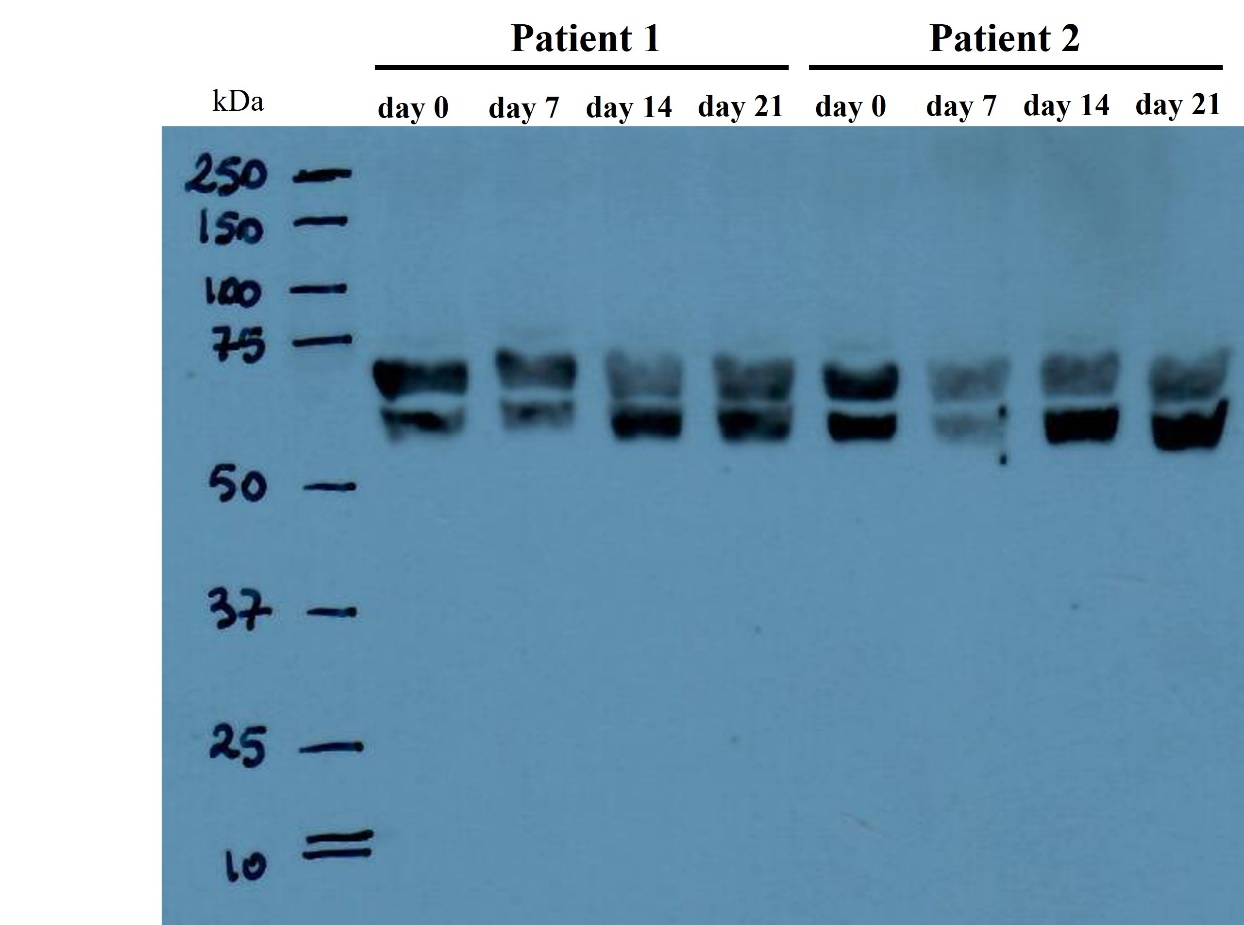


**Figure S4: Western blot image investigating Tenomodulin expression.**

Expression of the Tenomodulin protein extracted from the functionalized scaffold after 0,7,14, and 21 days of culture. N = 2 biological replicates.
